# Unilateral isometric contraction induces REDD1 and suppresses insulin‐stimulated mTORC1 and protein synthesis in non‐contracted muscle of male mice

**DOI:** 10.14814/phy2.70574

**Published:** 2025-09-19

**Authors:** Munkhtuul Munkh Amar, Taro Murakami

**Affiliations:** ^1^ Department of Nutrition Shigakkan University Obu Aichi Japan

**Keywords:** insulin, isometric contraction, mTORC, muscle protein synthesis, REDD1

## Abstract

Skeletal muscle hypertrophy is promoted by mechanical loading and is associated with activation of mTORC1 signaling. While REDD1, a stress‐responsive inhibitor of mTORC1, is typically downregulated in contracting muscle, we previously reported that unilateral isometric contraction increases REDD1 expression in the contralateral non‐contracted muscle. The functional significance of this response remains unclear. This study tested whether REDD1 induction in non‐contracted muscle attenuates anabolic signaling under insulin‐stimulated conditions. Male C57BL/6J mice underwent unilateral isometric contraction of the right gastrocnemius via percutaneous electrical stimulation. Insulin was administered systemically to stimulate mTORC1 signaling and protein synthesis. Western blotting was used to assess REDD1 protein levels and phosphorylation of mTORC1 downstream targets and Akt. REDD1 protein was significantly elevated in non‐contracted muscle following contraction. This was accompanied by reduced insulin‐stimulated phosphorylation of S6K1 and 4E‐BP1, as well as decreased puromycin incorporation, indicating suppressed protein synthesis. Insulin‐stimulated Akt phosphorylation was unchanged, suggesting that the suppression occurred downstream or independently of Akt. These findings demonstrate that isometric contraction can impair insulin‐stimulated mTORC1 signaling and protein synthesis in non‐contracted muscle in male mice, potentially via REDD1 induction.

## 1 INTRODUCTION

The progressive loss of skeletal muscle mass with aging and in chronic pathological conditions such as cancer and chronic obstructive pulmonary disease (COPD) is independently associated with increased mortality and reduced physiological resilience (Au et al., 2021; Chang et al., 2018; de Santana et al., 2021; Wang et al., 2023). Accordingly, elucidating the molecular mechanisms that regulate muscle hypertrophy is essential for developing effective strategies to preserve or restore muscle mass.

Both animal and human studies indicate that mechanical overload—typically achieved through resistance exercise—is the most effective stimulus for promoting skeletal muscle hypertrophy (reviewed in Roberts et al. (2023)). Acute bouts of mechanical loading transiently activate anabolic signaling pathways, most notably the mechanistic target of rapamycin complex 1 (mTORC1), resulting in increased rates of muscle protein synthesis (Baar & Esser, 1999; Ogasawara et al., 2016; Phillips et al., 1997). With repeated loading, these transient responses accumulate to drive long‐term muscle growth (Ogasawara et al., 2013).

mTORC1 activity is tightly regulated and can be inhibited by stress‐inducible factors such as REDD1 (Regulated in Development and DNA Damage Response 1), which is upregulated by glucocorticoids (Wang et al., 2003, 2006) and other catabolic signals (Brugarolas et al., 2004; DeYoung et al., 2008; Favier et al., 2010; Hayasaka et al., 2014; McGhee et al., 2009; Murakami et al., 2011; Shoshani et al., 2002; Sofer et al., 2005). Interestingly, while REDD1 is known to suppress mTORC1 activity, several studies have shown that its expression decreases in contracting muscle after resistance exercise in humans (Drummond et al., 2008) or following electrical stimulation in animals (Gordon et al., 2014), thereby facilitating anabolic signaling. In contrast, our recent work demonstrated that unilateral isometric contraction in mice induces a transient increase in REDD1 expression in the contralateral non‐contracted muscle, but not in the stimulated muscle (Murakami, 2023). This systemic response is at least partially mediated by glucocorticoid signaling. However, due to the absence of an anabolic stimulus in that study, no corresponding suppression of mTORC1 activity or protein synthesis was detected in non‐contracted muscle, leaving the functional relevance of REDD1 induction unresolved.

In the present study, we tested the hypothesis that REDD1 upregulation in non‐contracted muscle is sufficient to suppress mTORC1 signaling and protein synthesis under anabolic conditions. To evaluate this, insulin was administered after contraction to stimulate mTORC1 activity, and we assessed whether unilateral isometric contraction suppresses insulin‐stimulated anabolic signaling in the contralateral non‐contracted muscle. Our findings demonstrate that insulin‐induced increases in protein synthesis and mTORC1 signaling were suppressed by isometric contraction in non‐contracted muscle.

## 2 MATERIALS AND METHODS

### 2.1 Animals

Male C57BL/6J mice (9 weeks old; Japan SLC Inc., Hamamatsu, Japan) were acclimatized for 1 week under controlled conditions (22°C, 50% humidity, 12:12 h light–dark cycle) with ad libitum access to food (CE‐2; CLEA Japan) and water. All experimental procedures were approved by the Experimental Animal Care Committee of Shigakkan University. At 10 weeks of age, mice were randomly assigned to either an isometric contraction (IC) or resting (R) group. Each group was further divided into insulin (INS) or phosphate‐buffered saline (PBS) subgroups (*n* = 5 per subgroup).

### 2.2 Muscle contraction protocol

In the IC group, the gastrocnemius muscle was unilaterally subjected to isometric contraction as described by Maruyama et al. (2020). Briefly, under isoflurane anesthesia (2%–3% inhalation), the right gastrocnemius muscle was contracted isometrically via percutaneous electrical stimulation (100 Hz; five sets of ten 3‐s contractions with a 7‐s rest between contractions and a 3‐min rest between sets). The stimulation voltage (30 V) and frequency were adjusted to elicit maximal isometric tension, as previously described (Ashida et al., 2018; Zhao et al., 2017). The left gastrocnemius muscle served as the non‐contracted control. To minimize the influence of feeding‐induced anabolic signaling, mice were fasted for 3 h prior to the contraction protocol. Muscle samples were collected 3 h after contraction, the time point at which REDD1 induction was previously observed (Murakami, 2023).

### 2.3 Administration of insulin and puromycin

To stimulate mTORC1 signaling and muscle protein synthesis, anesthetized mice (2% isoflurane in oxygen) were retro‐orbitally administered insulin (0.5 U/kg body weight; #2492, Novolin R, Novo Nordisk Pharma) via the right orbital sinus in the INS groups at 2 h and 45 min after contraction. This dose was selected based on previous studies showing that 0.5–1.0 U/kg body weight is sufficient to activate Akt–mTORC1 signaling and enhance muscle protein synthesis in rodents (Dungana et al., 2014; Maruyama et al., 2020). Although this dose exceeds physiological postprandial insulin concentrations, it provides a robust anabolic stimulus suitable for detecting potential intermuscle cross talk effects on insulin responsiveness without the confounding influence of amino acid co‐administration. Mice in the PBS groups received an equivalent volume of phosphate‐buffered saline. Five minutes later (i.e., 2 h and 50 min post‐contraction), all mice were retro‐orbitally administered puromycin (40 μmol/kg body weight; #P8833, Sigma‐Aldrich) via the left orbital sinus to label newly synthesized peptides for subsequent analysis. Mice were euthanized by cervical dislocation exactly 10 min after puromycin administration (15 min after insulin administration), while still under anesthesia. The gastrocnemius muscle was rapidly excised, flash‐frozen in liquid nitrogen, and stored at −80°C until analysis.

### 2.4 Western blot

Western blotting, including the preparation of muscle extracts, was performed as previously described (Murakami, 2023). Briefly, protein extracts (25 μg) from the gastrocnemius muscle were separated by 10% SDS–polyacrylamide gel electrophoresis (SDS–PAGE) and transferred onto 0.45 μm polyvinylidene fluoride (PVDF) membranes (Millipore, Billerica, MA) using a semidry transfer system (Bio‐Rad Laboratories). Membranes were blocked for 1 h using Can Get Signal PVDF Blocking Reagent (Toyobo), then incubated overnight at 4°C with the following primary antibodies: phospho‐Thr389 S6K1 (#9234), total S6K1 (#2708), total 4E‐BP1 (#9644), phospho‐Thr308 Akt (#13038), phospho‐Ser473 Akt (#4060), and pan‐Akt (#4691) (Cell Signaling Technology); REDD1 (#10638‐1‐AP, Proteintech Group). All primary antibodies were diluted 1:4000 in Can Get Signal Solution 1 (#NKB‐20, Toyobo). Following incubation, membranes were washed with Tris‐buffered saline containing 0.1% Tween 20 (TBST) and then incubated with horseradish peroxidase–conjugated secondary antibodies (#1706515, Bio‐Rad Laboratories) diluted 1:10,000 in 3% skim milk/TBST for 1 h at room temperature. Protein bands were visualized using enhanced chemiluminescence detection reagents (ECL Prime Western Blotting Detection Reagent, #RPN2232, GE Healthcare UK) and imaged using the ImageQuant LAS500 system (GE Healthcare UK). Band intensities were quantified using ImageJ software (NIH). The phosphorylation levels of S6K1 (Thr389), Akt (Thr308 and Ser473) were normalized to the corresponding total protein signals and expressed relative to the mean value of the R‐PBS group. Phosphorylation of 4E‐BP1 was expressed as the percentage of the γ‐band relative to total 4E‐BP1 signal [γ/(α + β + γ) × 100]. REDD1 protein levels were normalized to Coomassie Brilliant Blue (CBB)‐stained total protein from the same membrane and expressed relative to the mean value of the R‐PBS group.

### 2.5 Protein synthesis

The rate of protein synthesis was measured by the SUnSET method (Schmidt et al., 2009) using an anti‐puromycin monoclonal antibody (#MABE343, Merck Millipore) and a secondary antibodies (Peroxidase AffiniPure Goat Anti‐Mouse IgG, Fcγ subclass 2a specific, #AB_2338514, Jackson ImmunoResearch Inc) as described previously (Hayasaka et al., 2014; Murakami, 2023).

### 2.6 Statistical analysis

To evaluate differences within the IC groups, data were analyzed using two‐way repeated measures analysis of variance (ANOVA). When a significant interaction was detected, paired (non‐contracted [N] vs. contracted [IC] muscle within a group) or unpaired (PBS‐IC vs. INS‐IC group) Student's *t*‐tests were performed as post hoc analyses. For comparisons involving non‐contracted muscles, two‐way factorial ANOVA (group × treatment) was employed. When a significant interaction was found, unpaired Student's *t*‐tests were conducted as post hoc analyses. A *p* value of less than 0.05 was considered statistically significant. Post hoc power analysis was conducted using G*Power 3.1, based on the observed effect sizes for REDD1 protein content and puromycin‐labeled peptide content, which were considered the primary outcomes of this study.

## 3 RESULTS

### 3.1 Isometric contraction increases REDD1 protein content in non‐contracted muscle

**FIGURE 1:**
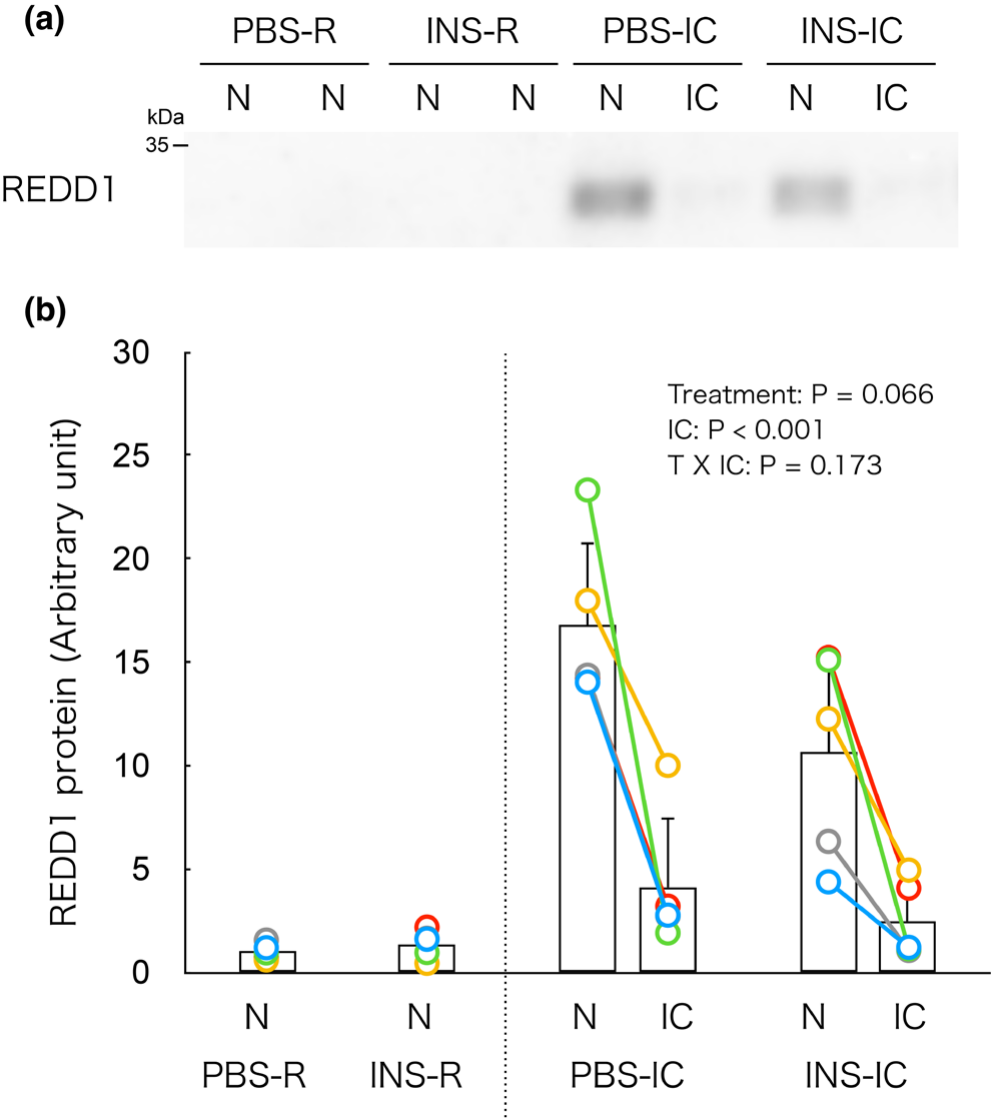
Isometric contraction increases REDD1 protein content in non‐contracted muscle. (a) Representative immunoblot of REDD1 protein. (b) Changes in REDD1 protein content by the isometric contraction. In the PBS‐R and INS‐R groups, REDD1 protein content showed no significant differences between the left and right muscles. Therefore, the average value of the left and right muscles is presented as a single bar with individual data points represented as dots in the figure. In the PBS‐IC and INS‐IC groups, each connected dot represents data obtained from the same animal. IC, isometrically contracted muscle; INS‐IC, mice treated with insulin and isometric contraction; INS‐R, mice treated with insulin (0.5 U/kg BW); N, non‐contracted muscle; PBS‐IC, mice treated with phosphate buffered saline and isometric contraction; PBS‐R, mice treated with phosphate‐buffered saline. The bars in the figure represent the mean ± SD, *n* = 5/group.

In the IC groups, two‐way ANOVA revealed a significant main effect of isometric contraction on REDD1 protein content, with no significant effect of insulin treatment or interaction between the two factors (Figure 1b). These findings indicate that isometric contraction increases REDD1 expression in non‐contracted muscle independently of insulin stimulation.

To determine whether this increase in REDD1 reflected upregulation in non‐contracted muscle or a relative decrease in the contralateral contracted muscle, we directly compared non‐contracted muscles from IC mice with those from R mice. Two‐way ANOVA revealed a significant main effect of isometric contraction and a significant interaction between the two factors (Figure S1 https://doi.org/10.6084/m9.figshare.29899997). Post hoc analysis showed that REDD1 protein content was significantly higher in the PBS‐IC group than in the PBS‐R group, and likewise significantly higher in the INS‐IC group than in the INS‐R group. Furthermore, REDD1 content in the INS‐IC group tended to be lower than in the PBS‐IC group (*p* = 0.065), suggesting that insulin may partially attenuate contraction‐induced REDD1 upregulation in non‐contracted muscle.

Post hoc power analysis revealed that the effect size for REDD1 (non‐contracted vs. contracted muscle in the INS‐IC group) was Cohen's *d* = 2.15, yielding an estimated statistical power (1−β) of 0.845 with *α* = 0.05 and *n* = 5 per group. This value exceeds the commonly accepted threshold of 0.8 for sufficient statistical power, indicating that the sample size was adequate to detect biologically meaningful differences.

### 3.2 Isometric contraction decreases insulin‐induced increase in protein synthesis in non‐contracted muscle

**FIGURE 2:**
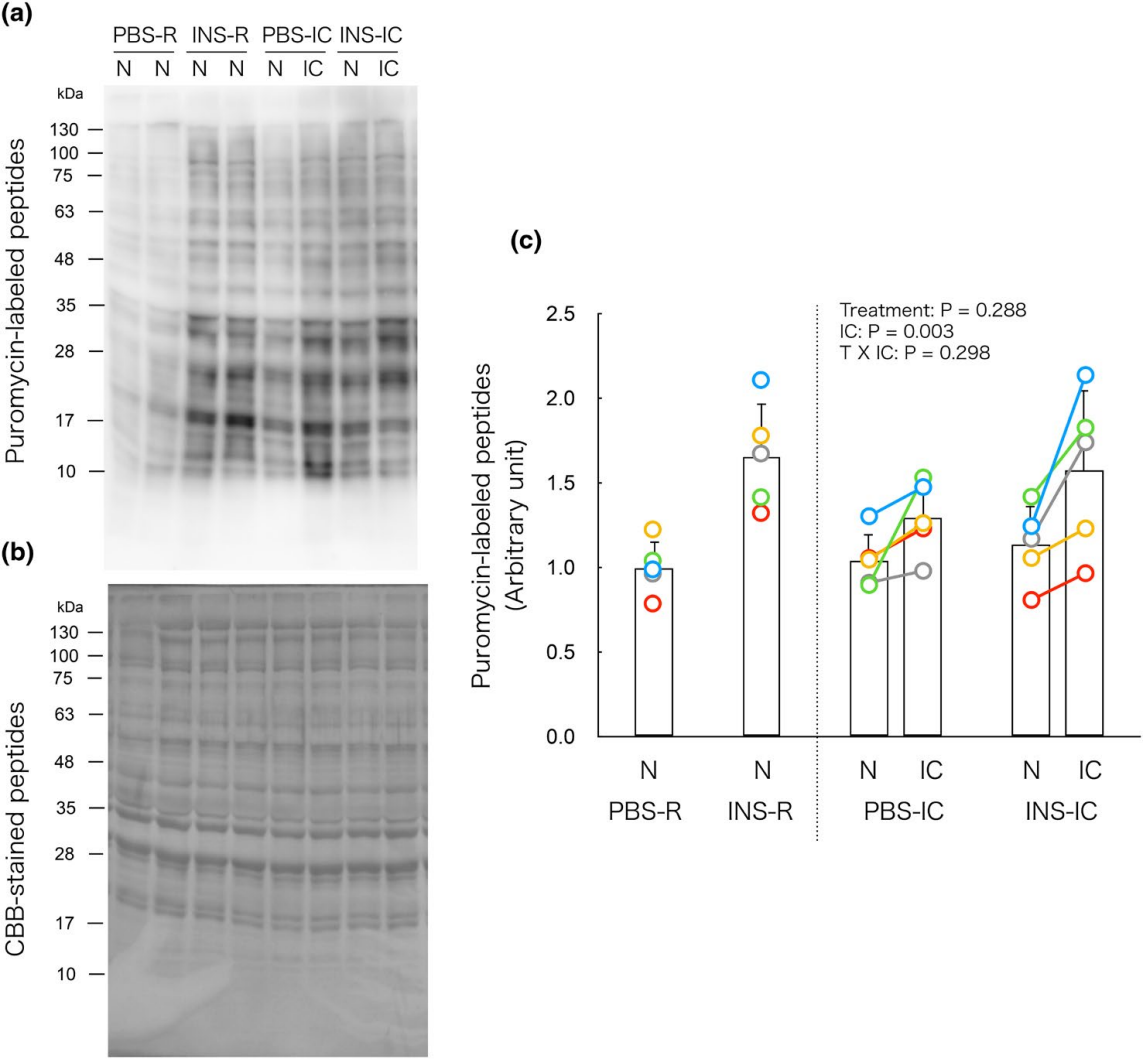
Isometric contraction decreases insulin‐induced increase in protein synthesis in non‐contracted muscle. (a) Representative immunoblot of puromycin‐labeled peptides. (b) Coomassie brilliant blue (CBB)‐stained whole‐lane protein. (c) Changes in puromycin‐labeled peptides by the insulin and/or isometric contraction. In the PBS‐R and INS‐R groups, puromycin‐labeled peptides showed no significant differences between the left and right muscle of each mouse. Therefore, the average value of the left and right muscles is presented as a single bar with individual data points represented as dots in the figure. In the PBS‐IC and INS‐IC groups, each connected dot represents data obtained from the same animal. IC, isometrically contracted muscle; INS‐IC, mice treated with insulin and isometric contraction; INS‐R, mice treated with insulin (0.5 U/kg BW); N, non‐contracted muscle; PBS‐IC, mice treated with phosphate buffered saline and isometric contraction; PBS‐R, mice treated with phosphate‐buffered saline. The bars in the figure represent the mean ± SD, *n* = 5/group.

In the IC groups, two‐way ANOVA revealed a significant main effect of isometric contraction on puromycin‐labeled peptide content, with no significant effect of insulin treatment or interaction between the two factors (Figure 2c). These results suggest that isometric contraction increases global protein synthesis in muscle, regardless of insulin stimulation.

To determine whether the observed increase reflected a true upregulation in contracted muscle or a relative decrease in non‐contracted muscle, we directly compared non‐contracted muscles between IC and R mice. Two‐way ANOVA revealed significant main effects of insulin treatment and isometric contraction, as well as a significant interaction (Figure S2 https://doi.org/10.6084/m9.figshare.29899997). Post hoc analysis showed that insulin significantly increased protein synthesis in the R group, but this effect was attenuated in the IC group. These findings indicate that isometric contraction suppresses insulin‐stimulated protein synthesis in non‐contracted muscle.

Post hoc power analysis revealed that the effect size for puromycin‐labeled peptides (non‐contracted muscle in the INS‐R group vs. non‐contracted muscle in the INS‐IC group) was Cohen's *d* = 1.91, yielding an estimated statistical power (1−β) of 0.753 with *α* = 0.05 and *n* = 5 per group. Although slightly below the conventional threshold of 0.8, this value indicates that the sample size provided adequate power to detect a biologically meaningful difference.

### 3.3 Isometric contraction decreases insulin‐induced increase in mTORC1 signaling in non‐contracted muscle

**FIGURE 3:**
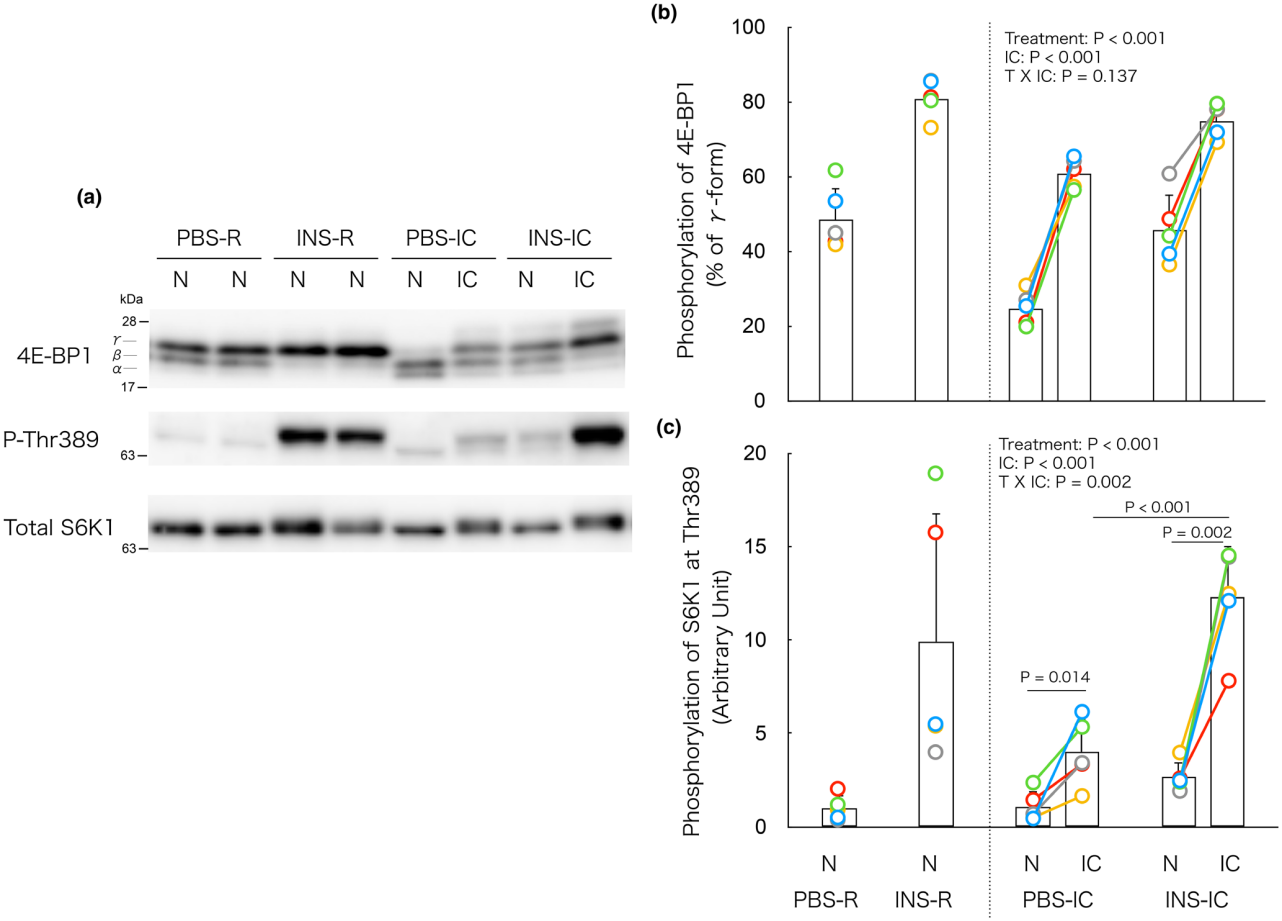
Isometric contraction decreases insulin‐induced increase in mTORC1 signaling in non‐contracted muscle. (a) Representative immunoblots of phosphorylation of 4E‐BP1 and S6K1 (T389), respectively. (b, c) Changes in phosphorylation of 4E‐BP1 and S6K1, respectively, by the insulin and/or isometric contraction. In the PBS‐R and INS‐R groups, phosphorylation of 4E‐BP1 and S6K1 showed no significant differences between the left and right muscles of each mouse. Therefore, the average value of the left and right muscles is presented as a single bar with individual data points represented as dots in the figure (b, c). In the PBS‐IC and INS‐IC groups, each connected dot represents data obtained from the same animal. IC, isometrically contracted muscle; INS‐IC, mice treated with insulin and isometric contraction; INS‐R, mice treated with insulin (0.5 U/kg BW); N, non‐contracted muscle; PBS‐IC, mice treated with phosphate buffered saline and isometric contraction; PBS‐R, mice treated with phosphate‐buffered saline. The bars in the figure represent the mean ± SD, *n* = 5/group. Specific *p* values for significant differences are shown above the horizontal bars.

In the IC groups, two‐way ANOVA revealed significant main effects of both insulin treatment and isometric contraction on 4E‐BP1 phosphorylation, with no significant interaction (Figure 3b). These findings indicate that insulin and contraction independently increase 4E‐BP1 phosphorylation in muscle. For S6K1 phosphorylation at Thr389, two‐way ANOVA revealed significant main effects of both factors as well as a significant interaction (Figure 3c). Post hoc analysis showed that S6K1 phosphorylation in the contracted muscle was significantly higher than in the non‐contracted muscle in both PBS‐IC and INS‐IC groups. Moreover, phosphorylation in the contracted muscle of the INS‐IC group was significantly higher than in the PBS‐IC group. These findings suggest a synergistic effect of insulin and contraction on S6K1 phosphorylation in muscle.

To evaluate whether isometric contraction suppresses insulin‐induced mTORC1 signaling in non‐contracted muscle, we compared non‐contracted muscles between IC and R mice (Figure S3a,b https://doi.org/10.6084/m9.figshare.29899997). For 4E‐BP1 phosphorylation, two‐way ANOVA revealed significant main effects of insulin and contraction, indicating that insulin increased and contraction decreased phosphorylation independently. In contrast, S6K1 phosphorylation showed both main effects and a significant interaction. Post hoc analysis revealed that insulin‐induced phosphorylation of S6K1 in the non‐contracted muscle of INS‐IC mice was significantly lower than in the INS‐R group. These findings indicate that isometric contraction attenuates insulin‐induced activation of mTORC1 signaling in non‐contracted muscle, particularly at the level of S6K1 phosphorylation.

### 3.4 Isometric contraction does not alter insulin‐induced increase in Akt phosphorylation in either contracted or non‐contracted muscle

**FIGURE 4:**
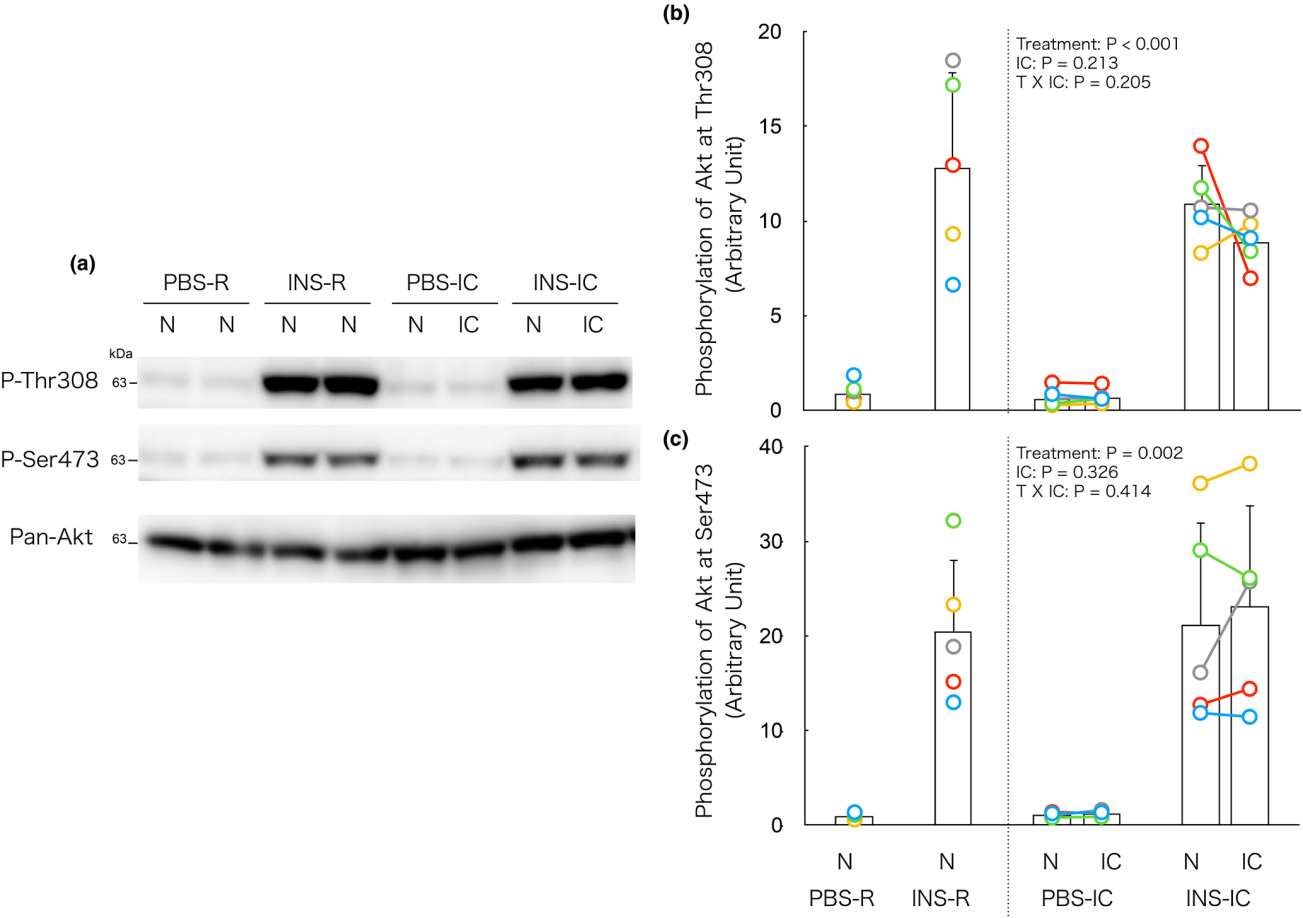
Isometric contraction does not alter insulin‐induced increase in Akt phosphorylation in both contracted and non‐contracted muscle. (a) Representative immunoblots of phosphorylation of Akt at Thr308 and Ser473. (b, c) Changes in phosphorylation of Akt at Thr308 and Ser473, respectively, by the insulin and/or isometric contraction. In the PBS‐R and INS‐R groups, phosphorylation of 4E‐BP1 and S6K1 showed no significant differences between the left and right muscles of each mouse. Therefore, the average value of the left and right muscles is presented as a single bar with individual data points represented as dots in the figure (b, c). In the PBS‐IC and INS‐IC groups, each connected dot represents data obtained from the same animal. IC, isometrically contracted muscle; INS‐IC, mice treated with insulin and isometric contraction; INS‐R, mice treated with insulin (0.5 U/kg BW); N, non‐contracted muscle; PBS‐IC, mice treated with phosphate buffered saline and isometric contraction; PBS‐R, mice treated with phosphate‐buffered saline. The bars in the figure represent the mean ± SD, *n* = 5/group.

In the IC groups, two‐way ANOVA revealed significant main effects of insulin treatment on Akt phosphorylation at both Thr308 and Ser473 (Figure 4b,c), with no significant effects of isometric contraction or interaction. These findings indicate that insulin increases Akt phosphorylation independently of isometric contraction.

To determine whether insulin‐induced Akt phosphorylation in non‐contracted muscle is affected by isometric contraction, we directly compared non‐contracted muscles between IC and R groups (Figure S4a,b https://doi.org/10.6084/m9.figshare.29899997). Two‐way ANOVA again revealed significant main effects of insulin treatment at both phosphorylation sites, without significant effects of contraction or interaction. These results suggest that isometric contraction does not influence insulin‐induced Akt phosphorylation in non‐contracted muscle. Together, these findings indicate that Akt phosphorylation in response to insulin is unaffected by isometric contraction in either contracted or non‐contracted muscle.

## 4 DISCUSSION

The present study demonstrates that unilateral isometric contraction suppresses insulin‐induced activation of mTORC1 signaling and protein synthesis in the contralateral non‐contracted muscle in male mice. This suppression was accompanied by a significant increase in REDD1 protein content in non‐contracted muscle. These findings support the hypothesis that contraction‐induced systemic signals, likely mediated by glucocorticoids, inhibit anabolic signaling in non‐stimulated muscles via REDD1 upregulation.

Several studies have reported that REDD1 is upregulated by stress‐related stimuli, including glucocorticoids (Wang et al., 2003, 2006), hypoxia (Brugarolas et al., 2004; DeYoung et al., 2008; Favier et al., 2010; Shoshani et al., 2002), and energy depletion (McGhee et al., 2009; Sofer et al., 2005). In our study, unilateral contraction increased REDD1 protein content selectively in non‐contracted muscle, in agreement with previous findings (Murakami, 2023). The mechanism may involve systemic hormonal responses, particularly glucocorticoids, which are elevated following intense muscular activity and likely contribute to REDD1 upregulation. In our previous work using the same contraction protocol, plasma corticosterone concentrations measured at 3 h post‐contraction had already returned to baseline, with no difference between contracted and non‐contracted conditions (Murakami, 2023). However, administration of the glucocorticoid receptor antagonist RU‐486 markedly attenuated the contraction‐induced increase in REDD1 mRNA and protein content in non‐contracted muscle (Murakami, 2023). These findings suggest that transient elevations in circulating glucocorticoids, possibly occurring earlier than 3 h post‐contraction, could mediate REDD1 induction in non‐contracted muscle, although the involvement of direct transcriptional regulation remains to be determined.

Conversely, resistance exercise in humans and electrically stimulated contraction in rodents has been shown to reduce REDD1 expression in contracting muscle, respectively (Drummond et al., 2008; Gordon et al., 2014). Indeed, in the current study, REDD1 expression should be reduced in contracted muscle. This downregulation may facilitate mTORC1 activation by relieving REDD1‐mediated inhibition. Although direct evidence is limited, Akt has been shown to phosphorylate and inhibit FoxO transcription factors (Brunet et al., 1999), which in turn have been implicated in the transcriptional activation of REDD1 (Harvey et al., 2008), suggesting a possible mechanism by which Akt signaling could suppress REDD1 expression. While it has been argued that translational regulation may predominate over this short time frame (i.e., <3 h), our previous work demonstrated a significant increase in REDD1 mRNA levels in non‐contracted muscle within 3 h following unilateral contraction (Murakami, 2023), indicating that transcriptional regulation of REDD1 can occur within this time window. However, we did not observe increased Akt phosphorylation in contracted muscle under our isometric protocol. Notably, previous work has shown that contraction can increase Akt phosphorylation without requiring Akt for mTORC1 activation or protein synthesis (Maruyama et al., 2020), suggesting that REDD1 suppression in contracting muscle may occur via Akt‐independent mechanisms. Thus, while REDD1 is locally suppressed in contracting muscle to favor anabolic signaling, it is transiently upregulated in non‐contracted muscle, potentially reflecting a systemic regulatory response.

The physiological relevance of transient REDD1 upregulation in non‐contracted muscle remains speculative. One possible interpretation is that REDD1 acts as a systemic brake to prevent anabolic signaling in muscles that are not actively recruited during exercise, thereby prioritizing energy and nutrient availability for the contracting muscles. Supporting the concept that muscle activity can modulate metabolism in distant muscles, a recent human study demonstrated that one‐legged exercise to exhaustion acutely impaired insulin‐stimulated glucose uptake in the contralateral, non‐exercised leg, resulting in reduced whole‐body insulin sensitivity (Steenberg et al., 2020). It should be noted that the insulin administration protocol used in this study was supraphysiological and did not include coadministration of amino acids. Although this approach has been shown to robustly stimulate Akt–mTORC1 signaling and protein synthesis in rodents (Dungana et al., 2014; Maruyama et al., 2020), the absence of concurrent aminoacidemia may have influenced the anabolic response, particularly in the non‐contracted muscle. Moreover, we cannot exclude the possibility that insulin, by promoting amino acid uptake into the contracted muscle, may have reduced amino acid availability in the non‐contracted muscle, potentially attenuating protein synthesis there. Plasma amino acid concentrations were not measured in the present study, limiting further interpretation regarding systemic nutrient availability. These limitations should be taken into account when extrapolating the present findings to physiological postprandial conditions in humans, although the insulin‐only model remains valuable for mechanistically isolating the effect of contraction on insulin responsiveness.

Given that REDD1 expression in non‐contracted muscle peaks within a few hours after contraction (Murakami, 2023), the present findings likely reflect a transient systemic response that limits anabolic signaling in inactive muscle during and shortly after unilateral activity. This temporal pattern suggests that the observed suppression of mTORC1 signaling and protein synthesis may serve as a short‐term regulatory mechanism rather than a sustained inhibition.

In conclusion, this study reveals that unilateral isometric contraction is sufficient to inhibit insulin‐stimulated protein synthesis and mTORC1 signaling in non‐contracted muscle in male mice, likely through REDD1 upregulation. These findings identify REDD1 as a potential mediator of intermuscular cross talk during exercise and suggest new targets for preserving muscle mass under asymmetric loading or disuse conditions.

## AUTHOR CONTRIBUTIONS

M.M.A. and T.M. conceived and designed research; M.M.A. and T.M. performed experiments; M.M.A. analyzed data; M.M.A. and T.M. interpreted results of experiments; M.M.A. and T.M. prepared figures; M.M.A. and T.M. drafted manuscript; M.M.A. and T.M. edited and revised manuscript; M.M.A. and T.M. approved final version of manuscript.

## FUNDING INFORMATION

This work was supported by JSPS KAKENHI Grant Number JP22K11620 to TM.

## CONFLICT OF INTEREST STATEMENT

We have no conflict of interest concerning this research.

## ETHICS STATEMENT

All experimental procedures were approved by the Experimental Animal Care Committee of Shigakkan University.

## Supporting information


Figures S1–S4.

## Data Availability

The data that support the findings of this study are available from the corresponding author upon reasonable request.
