# Supplementary material for: Unilateral isometric contraction induces REDD1 and suppresses insulin‐stimulated mTORC1 and protein synthesis in non‐contracted muscle of male mice
Source: Physiol Rep. 2025 Sep 19;13(18):e70574. doi: 10.14814/phy2.70574 (PMC12446998; doi:10.14814/phy2.70574)
Supplement: Supplementary file 1 — Figures S1–S4. [file PHY2-13-e70574-s001.zip › PHYSREP-2025-06-580-T-s03.pdf]

Supplementary Figure 1
Isometric contraction increases REDD1 protein content in non-contracted muscle.
To investigate whether the increased REDD1 protein content in non-contracted muscles of the IC group was due to upregulation in these muscles or downregulation in contracted muscles, we directly compared non-contracted muscles from IC mice with muscles from rested (R) mice. Two-way ANOVA revealed a significant main effect of isometric contraction, as well as a significant interaction between treatment and contraction. REDD1 protein content in the non-contracted muscle of the INS-IC group tended to be lower than that of the PBS-IC group (P = 0.065). These results suggest that REDD1 expression was upregulated by isometric contraction in non-contracted muscle, and that this upregulation may be attenuated by insulin treatment. N, non-contracted muscle; INS-R, mice treated with insulin (0.5 U/kg BW); PBS-R, mice treated with phosphate-buffered saline; INS-IC, mice treated with insulin and isometric contraction; PBS-IC, mice treated with phosphate-buffered saline and isometric contraction. Data are presented as means ± SD (n = 5/group). Vertical bars with P values indicate post hoc comparisons following two-way ANOVA.

Supplementary Figure 2
Isometric contraction attenuates insulin-induced protein synthesis in non-contracted muscle.
To determine whether the increase in puromycin-labeled peptide content in contracted muscles of the IC group reflected reduced incorporation in non-contracted muscles, we directly compared non-contracted muscles from IC mice with muscles from rested (R) mice. Two-way ANOVA revealed significant main effects of treatment (PBS vs. insulin) and isometric contraction, along with a significant interaction. Puromycin-labeled peptide content in the INS-R group was significantly higher than that in the PBS-R group, confirming the anabolic effect of insulin. Notably, the content in non-contracted muscles of the INS-IC group was significantly lower than that in the INS-R group, indicating that insulin-induced protein synthesis was attenuated in non-contracted muscles by isometric contraction. N, non-contracted muscle; INS-R, mice treated with insulin (0.5 U/kg BW); PBS-R, mice treated with phosphate-buffered saline; INS-IC, mice treated with insulin and isometric contraction; PBS-IC, mice treated with phosphate-buffered saline and isometric contraction. Data are presented as means ± SD (n = 5/group). Vertical bars with P values indicate post hoc comparisons following two-way ANOVA.

Supplementary Figure 3
Isometric contraction reduces insulin-induced mTORC1 signaling in non-contracted muscle.
To determine whether mTORC1 signaling is suppressed in non-contracted muscles following unilateral isometric contraction, we directly compared phosphorylation of 4E-BP1 and S6K1 between non-contracted muscles from IC mice and muscles from rested (R) mice. Two-way ANOVA revealed significant main effects of treatment and isometric

contraction for 4E-BP1 phosphorylation, indicating that insulin increased and isometric contraction decreased phosphorylation independently. For S6K1, significant main effects and a significant interaction were observed. Insulin treatment significantly increased S6K1 phosphorylation in both R and IC groups; however, the phosphorylation level in non-contracted muscles of the INS-IC group was significantly lower than that in the INS-R group. These results indicate that insulin-induced phosphorylation of mTORC1 downstream targets was attenuated by isometric contraction in non-contracted muscle. N, non-contracted muscle; INS-R, mice treated with insulin (0.5 U/kg BW); PBS-R, mice treated with phosphate-buffered saline; INS-IC, mice treated with insulin and isometric contraction; PBS-IC, mice treated with phosphate-buffered saline and isometric contraction. Data are presented as means ± SD (n = 5/group). Vertical bars with P values indicate post hoc comparisons following two-way ANOVA.

Supplementary Figure 4

Isometric contraction does not alter insulin-induced Akt phosphorylation in non-contracted muscle.

To determine whether insulin-induced Akt phosphorylation is affected in non-contracted muscles following unilateral isometric contraction, we directly compared phosphorylation at Thr308 and Ser473 between non-contracted muscles from IC mice and muscles from rested (R) mice. Two-way ANOVA revealed significant main effects of treatment (PBS vs. insulin) for both phosphorylation sites, with no main effect of isometric contraction and no interaction. These findings indicate that insulin-induced Akt activation occurred similarly in both groups and was not altered in non-contracted muscle by isometric contraction. N, non-contracted muscle; INS-R, mice treated with insulin (0.5 U/kg BW); PBS-R, mice treated with phosphate-buffered saline; INS-IC, mice treated with insulin and isometric contraction; PBS-IC, mice treated with phosphate-buffered saline and isometric contraction. Data are presented as means ± SD (n = 5/group). Vertical bars with P values indicate post hoc comparisons following two-way ANOVA.
