# Supplementary figures and images for: Unilateral isometric contraction induces REDD1 and suppresses insulin‐stimulated mTORC1 and protein synthesis in non‐contracted muscle of male mice
Source: Physiol Rep. 2025 Sep 19;13(18):e70574. doi: 10.14814/phy2.70574 (PMC12446998; doi:10.14814/phy2.70574)

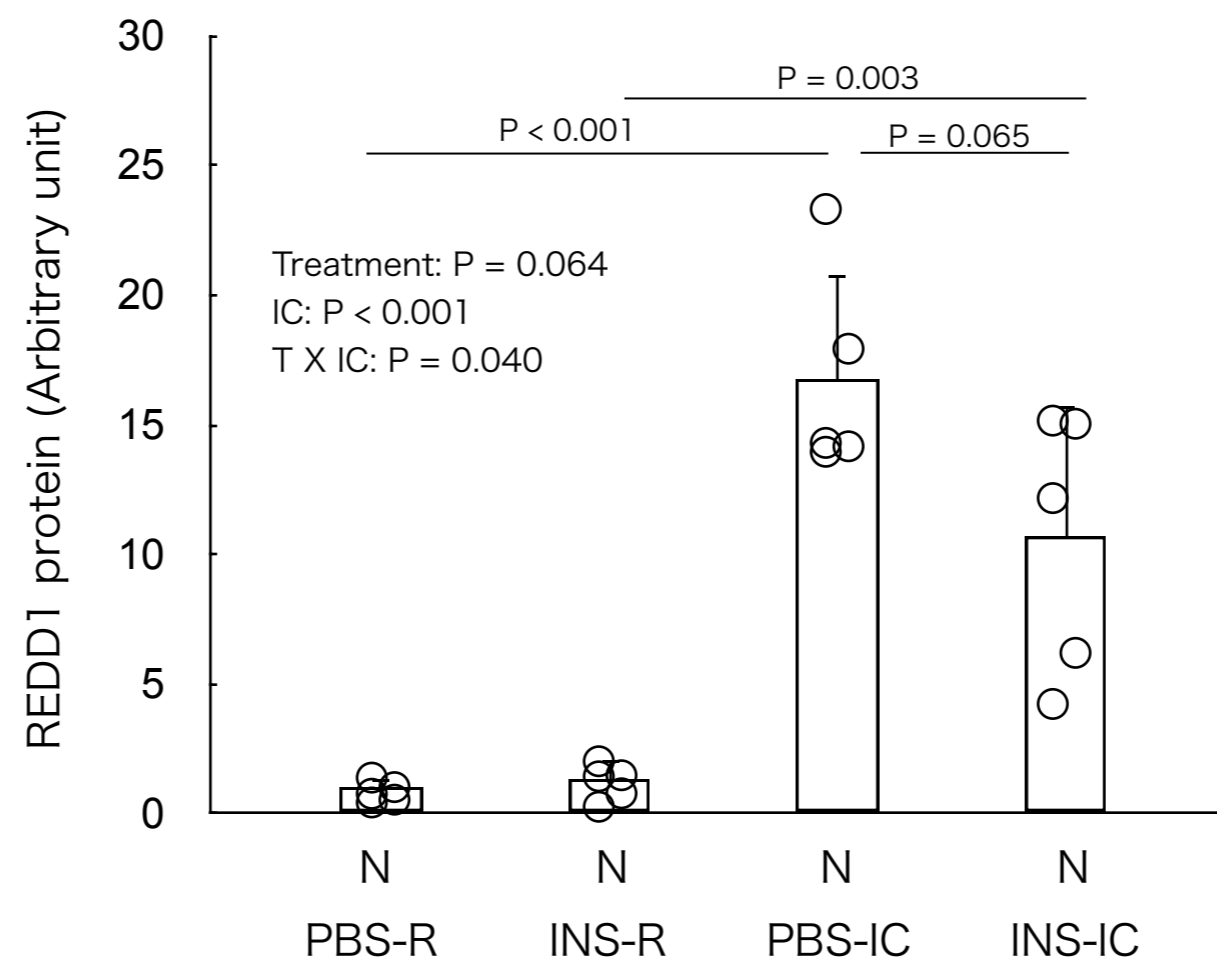

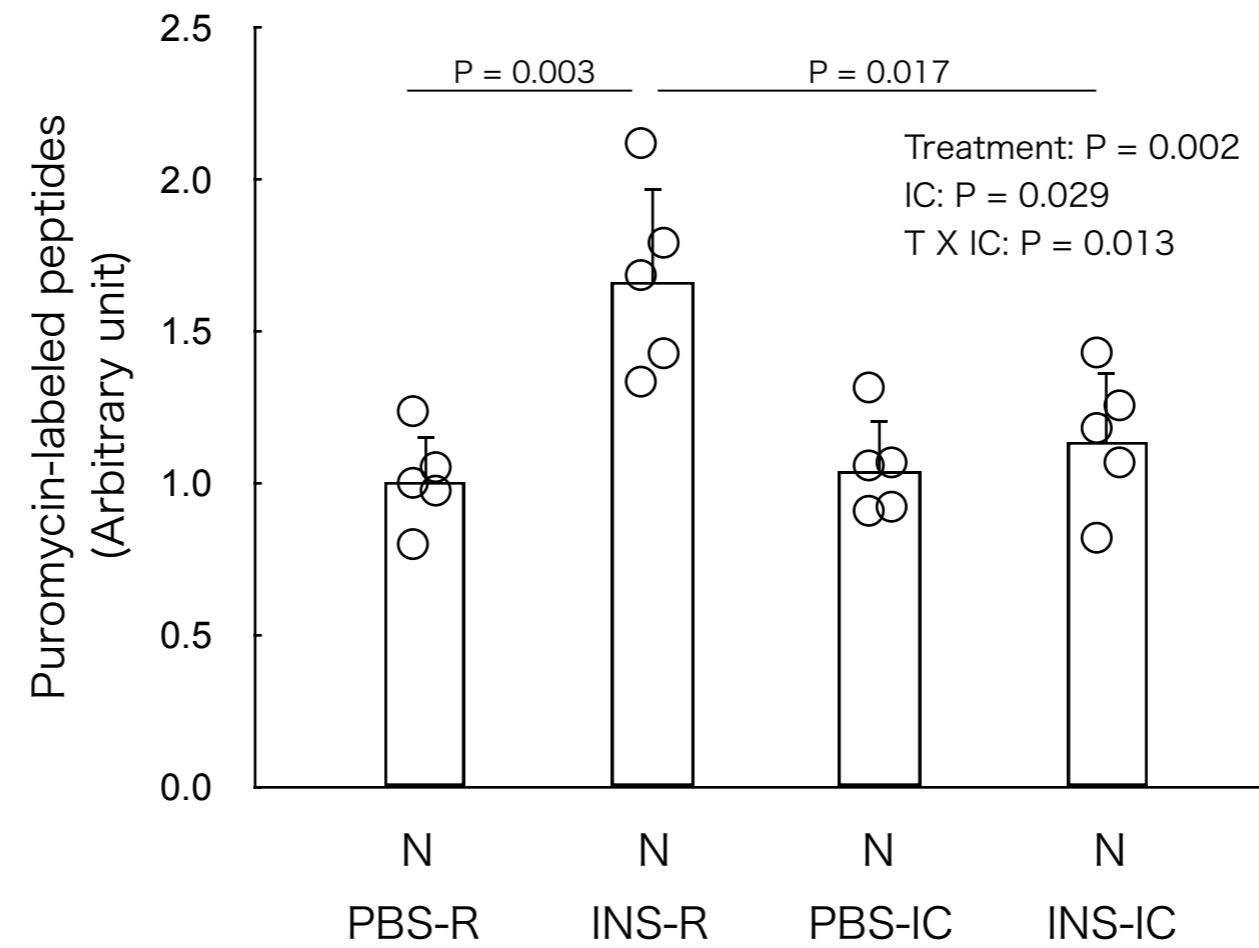

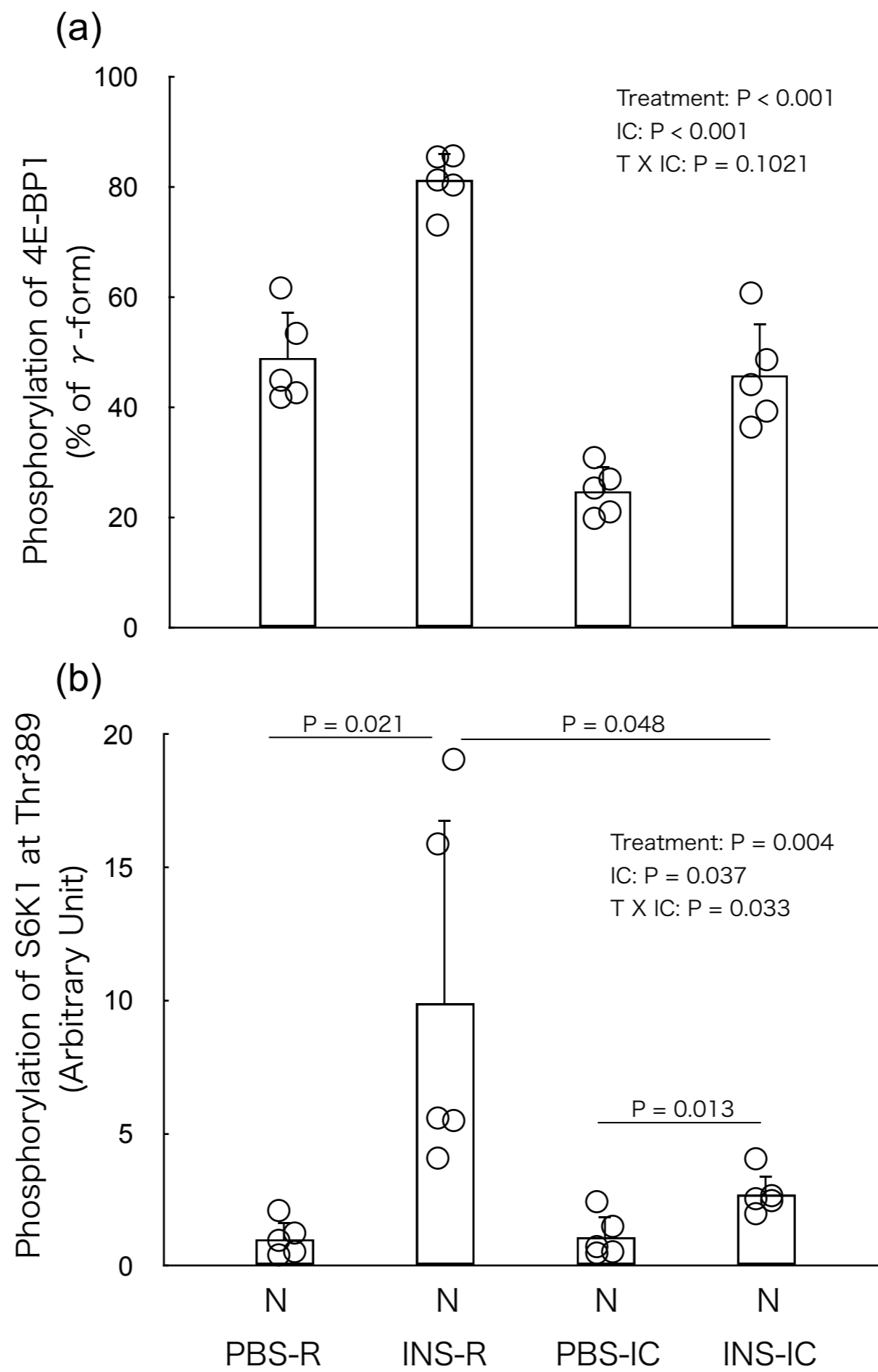

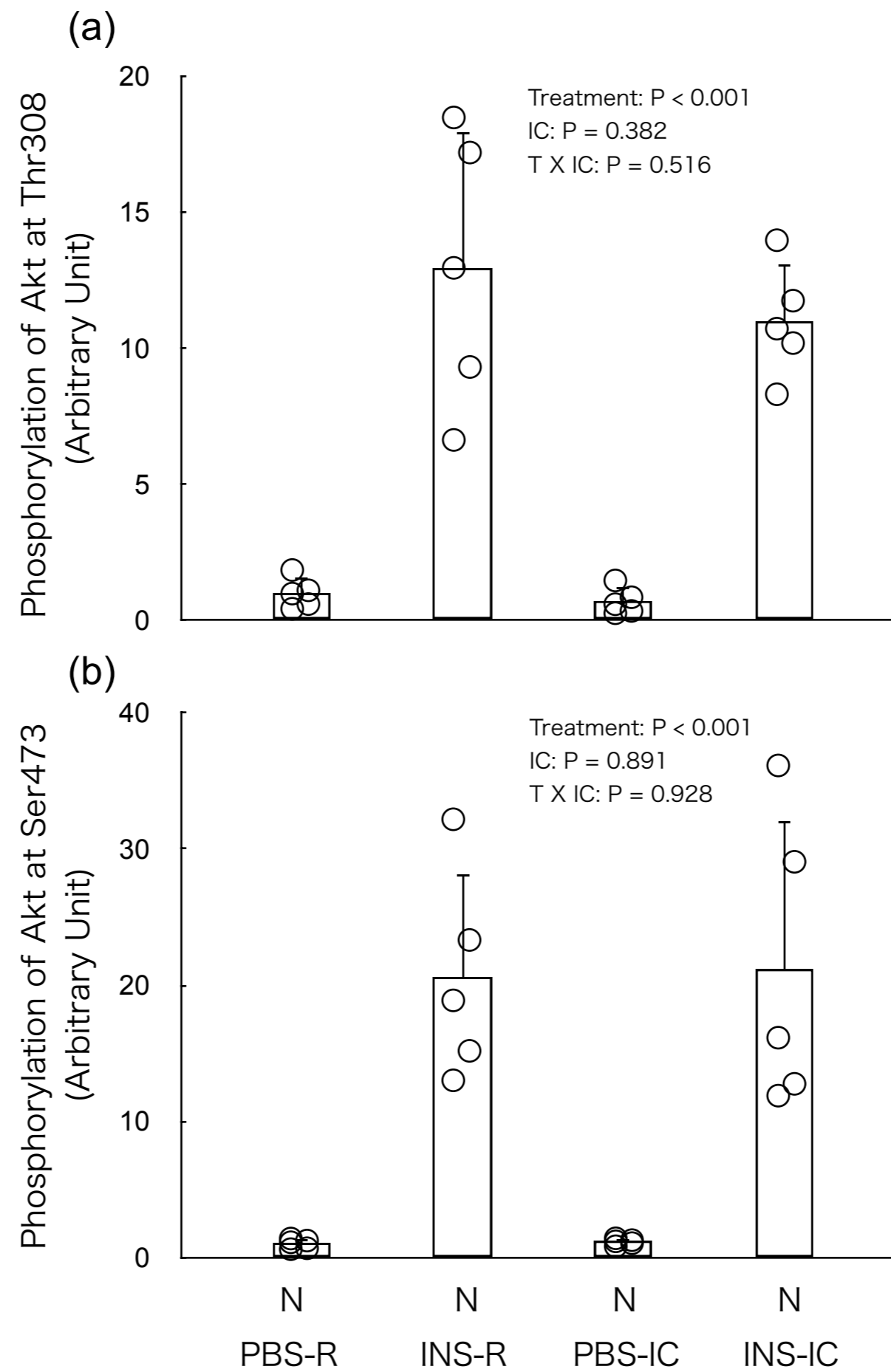

Supplement: Supplementary file 1 — Figures S1–S4. [file PHY2-13-e70574-s001.zip › PHYSREP-2025-06-580-T-s02.pdf]
